# Human Milk Oligosaccharide 2′-Fucosyllactose Inhibits Ligand Binding to C-Type Lectin DC-SIGN but Not to Langerin

**DOI:** 10.3390/ijms232314745

**Published:** 2022-11-25

**Authors:** Reshmi Mukherjee, Victor J. Somovilla, Fabrizio Chiodo, Sven Bruijns, Roland J. Pieters, Johan Garssen, Yvette van Kooyk, Aletta D. Kraneveld, Jeroen van Bergenhenegouwen

**Affiliations:** 1 Division of Pharmacology, Utrecht Institute of Pharmaceutical Sciences, Faculty of Science, Utrecht University, Universiteitsweg 99, 3508 TB Utrecht, The Netherlands; 2 Division of Chemical Biology and Drug Discovery, Utrecht Institute of Pharmaceutical Sciences, Faculty of Science, Utrecht University, Universiteitsweg 99, 3508 TB Utrecht, The Netherlands; 3 Center for Cooperative Research in Biomaterials (CIC biomaGUNE), Basque Research and Technology Alliance (BRTA), Paseo de Miramon 182, 20014 Donostia San Sebastián, Spain; 4 Department of Molecular Cell Biology and Immunology, Amsterdam UMC, Location Vrije Universiteit, Amsterdam Infection and Immunity Research Institute, De Boelelaan 1108, 1081 HZ Amsterdam, The Netherlands; 5 Danone Nutricia Research, Uppsalalaan 12, 3584 CT Utrecht, The Netherlands

**Keywords:** DC-SIGN, 2′-FL, human milk oligosaccharides, langerin, lewis B antigen

## Abstract

Human milk oligosaccharides (HMOs) and their most abundant component, 2′-Fucosyllactose (2′-FL), are known to be immunomodulatory. Previously, it was shown that HMOs and 2′-FL bind to the C-type lectin receptor DC-SIGN. Here we show, using a ligand-receptor competition assay, that a whole mixture of HMOs from pooled human milk (HMOS) and 2′-FL inhibit the binding of the carbohydrate-binding receptor DC-SIGN to its prototypical ligands, fucose and the oligosaccharide Lewis-B, (Le^b^) in a dose-dependent way. Interestingly, such inhibition by HMOS and 2′-FL was not detected for another C-type lectin, langerin, which is evolutionarily similar to DC-SIGN. The cell-ligand competition assay using DC-SIGN expressing cells confirmed that 2′-FL inhibits the binding of DC-SIGN to Le^b^. Molecular dynamic (MD) simulations show that 2′-FL exists in a preorganized bioactive conformation before binding to DC-SIGN and this conformation is retained after binding to DC-SIGN. Le^b^ has more flexible conformations and utilizes two binding modes, which operate one at a time via its two fucoses to bind to DC-SIGN. Our hypothesis is that 2′-FL may have a reduced entropic penalty due to its preorganized state, compared to Le^b^, and it has a lower binding enthalpy, suggesting a better binding to DC-SIGN. Thus, due to the better binding to DC-SIGN, 2′-FL may replace Le^b^ from its binding pocket in DC-SIGN. The MD simulations also showed that 2′-FL does not bind to langerin. Our studies confirm 2′-FL as a specific ligand for DC-SIGN and suggest that 2′-FL can replace other DC-SIGN ligands from its binding pocket during the ligand-receptor interactions in possible immunomodulatory processes.

## 1. Introduction

The C-type lectin receptor (CLR) family are pattern recognition receptors that play an important role in maintaining the host tissue homeostasis but are also critically important in host-microbe interactions [1] The family of CLRs consists of many different subgroups harbouring several different receptors that all share a calcium-dependent carbohydrate-recognition domain (CRD) [2]. CLRs respond to a wide variety of ligands, both from endogenous and exogenous origins, as (glyco)lipids, (glyco)proteins, or glycans [3].

Two of the best-studied CLRs are dendritic cell-specific intercellular adhesion molecule-3-grabbing non-integrin (DC-SIGN) and langerin which recognize various glycan motifs [4]. DC-SIGN is expressed by immature dendritic cells from the dermis, mucosal tissues, and lymph nodes [5]. Langerin is expressed by Langerhans cells, a subset of immature dendritic cells found in the epidermis of the human skin, lung, kidney, and liver [6]. DC-SIGN and langerin both have a glutamic acid-proline-asparagine (EPN) amino acid binding motif in their CRDs and use that motif to interact with fucose monosaccharide as well as fucose-containing motifs, such as Lewis-type antigens Lewis -X (Le^x^), Lewis-Y (Le^y^), Lewis-A (Le^a^), and Lewis-B (Le^b^). In most cases, DC-SIGN recognizes all four Lewis antigens [7,8]; however, langerin recognizes mostly Le^b^ and Le^Y^ [4,9].

Human milk oligosaccharides (HMOs) are abundant in human breast milk. HMOs contain mostly lactose (~70 g/L) and other various complex glycan structures (5–20 g/L) that are derived from lactose [10,11,12]. In the last decade, interest in HMOs has surged and several health benefits have been attributed to them. HMOs are primarily seen as prebiotic substrates for beneficial bacteria, but accumulating research suggests that HMOs also have anti-pathogenic properties [13]. Moreover, HMOs have been found to directly interact with host cells promoting gut health by improving the epithelial barrier function [14,15], but also by the direct immunomodulating effects, such as the activation or inhibition of Toll-like receptor (TLR) signalling [16,17]. However, the exact mechanism of action of direct immunomodulation by HMOs remains to be determined.

2′-fucosyllactose (2′-FL) is one of the most abundant HMOs and has also been shown to exert a direct effect on host immune cells or tissues, as well as indirect effects by acting as a decoy receptor to inhibit the adhesion of pathogens [18,19,20,21,22,23]. Currently, 2′-FL can be produced via different methods. In the past, 2′-FL was chemically synthesized from lactose, while recently, 2′-FL is biotechnologically produced on a large scale [24].

To explain the immunomodulatory functions of HMOs, researchers have investigated carbohydrate-binding receptors for HMO binding. Using glycan-microarrays, it was shown that HMOs specifically interact with different lectins, based on their structure and affinity [25] 2′-FL was shown to specifically interact with DC-SIGN, but not with other CLRs [25].

**Figure 1:**
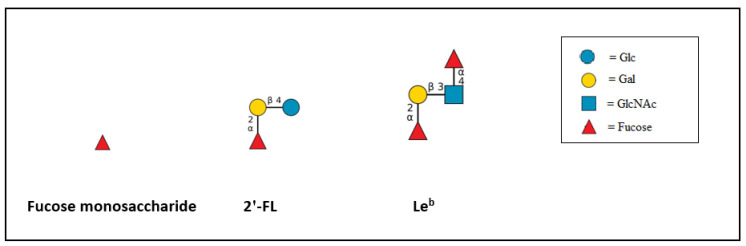
The schematic diagram of fucose monosaccharide, 2′-FL, and the terminal tetrasaccharide of the Le^b^ antigen are shown. Glc = glucose, Gal = galactose, GlcNAc = N-acetylglucosamine. The structures are drawn using DrawGlycan [26].

Here, we aim to study the effect of 2′-FL on the binding of DC-SIGN and langerin to their prototypical ligands fucose monosaccharide and Le^b^, whose structure is depicted below (Figure 1). In addition, we investigated the effect of three different preparations of 2′-FL, biotechnologically produced commercially available 2′-FL (c-2′-FL), purified c-2′-FL (p2′-FL), and chemically synthesized 2′-FL (s2′-FL) on the binding of those prototypical ligands to DC-SIGN. Molecular dynamic (MD) simulations were run to understand the molecular details and dynamics of the interaction of 2′-FL with DC-SIGN and langerin. In addition, the MD simulation was performed for the interaction of Le^b^ with DC-SIGN to understand whether 2′-FL might be able to displace Le^b^ from the CRD of DC-SIGN.

## 2. Results

### 2.1. c2′-FL, p2′-FL, s2′-FL and HMOs Inhibit the Ligand Binding to DC-SIGN, but Not to Langerin

**Figure 2:**
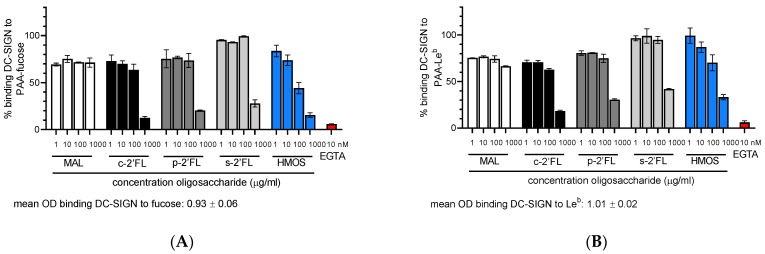

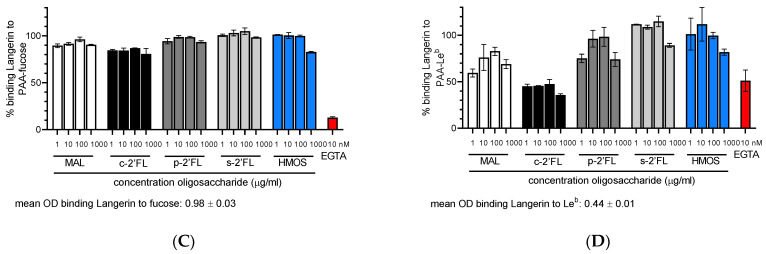
Ligand-receptor competition assay for the DC-SIGN-Fc and langerin-Fc (5 µg/mL) binding to PAA-fucose or PAA-Le^b^ with maltotriose (MAL, negative control), c2′-FL, p2′-FL, s2′-FL and HMOS [in various concentrations of 1 µg/mL, 10 µg/mL, 100 µg/mL, and 1000 µg/mL ]. EGTA [10 nM] was used to demonstrate the Ca^+2^-and Mg^+2^ dependent binding of DC-SIGN and langerin to the PAA-fucose and PAA-Le^b^ ligands. (**A**) inhibition by 2′-FLs and HMOS of the binding of DC-SIGN-Fc (5 µg/mL) to the ligand PAA-fucose; (**B**) inhibition by 2′-FLs and HMOS of the binding of DC-SIGN-Fc (5 µg/mL) to the ligand PAA-Le^b^; (**C**) No effect of 2′-FLs and HMOS on the binding of langerin-Fc (5 µg/mL) to the ligand PAA-fucose; (**D**) No effect of 2′-FLs and HMOS on the binding of langerin-Fc (5 µg/mL) to the ligand PAA-Le^b^. Results are presented as the mean % binding of the ligand to DC-SIGN-Fc and langerin-Fc ± SD, data from two separate experiments, performed in duplicate (n = 4 technical replicates). No block condition is regarded as 100% ligand-DC-SIGN-Fc/langerin-Fc binding. c-2′-FL = biotechnologically produced commercially available 2′-FL, p2′-FL = purified c-2′-FL and s2′-FL = chemically synthesized 2′-FL.

A ligand-receptor competition assay (solid-phase assay ELISA) was performed using a commercially available multivalent polyacrylamide (PAA)-linked fucose monosaccharide (PAA-fucose) or PAA-linked-Le^b^ (PAA-Le^b^), as ligands. These were used as coating agents for these experiments. The CLR-Fc constructs of DC-SIGN and langerin were used as receptors. Maltotriose (MAL) was used as a negative control. EGTA binds to the Ca^+2^ ion and thus it was used for inhibition of the CLR-carbohydrate binding. To investigate whether the side-products in the biotechnologically produced 2′-FL preparations (c2′-FL) play a role in affecting the ligand-CLR binding, we compared c2′-FL with purified c2′-FL (p2′-FL) and the chemically synthesized 2′-FL (s2′-FL). As a binding control, a whole mixture of HMOs isolated from pooled human milk (HMOS) was used. c2′-FL, p2′-FL, s2′-FL and HMOS were used at a concentration of 1 µg/mL, 10 µg/mL, 100 µg/mL, and 1000 µg/mL. c2′-FL, p2′-FL, s2′-FL and HMOS inhibited the binding of DC-SIGN-Fc (used at 5 µg/mL) to the ligand, PAA-Fucose and PAA- Le^b^ in a dose-dependent way (Figure 2A,B). The inhibition occurred for all of the 2′-FLs and HMOS, only at the 1000 µg/mL (1 mg/mL, 2 mM for 2′-FLs) concentration. On the contrary, c2′-FL, p2′-FL, s2′-FL, and HMOS did not inhibit the binding of langerin-Fc to the ligands PAA-fucose or PAA-Le^b^ (Figure 2C,D). When the assay was performed with a lower concentration of DC-SIGN-Fc (0.5 µg/mL and 0.1 µg/mL), a similar effect on the binding of DC-SIGN by all of the 2′-FLs and HMOS was observed (Figure S1A–D).

### 2.2. c2′-FL, p2′-FL, and s2′-FL Inhibit the Binding of Le^b^ to the DC-SIGN Expressing Cells

**Figure 3:**
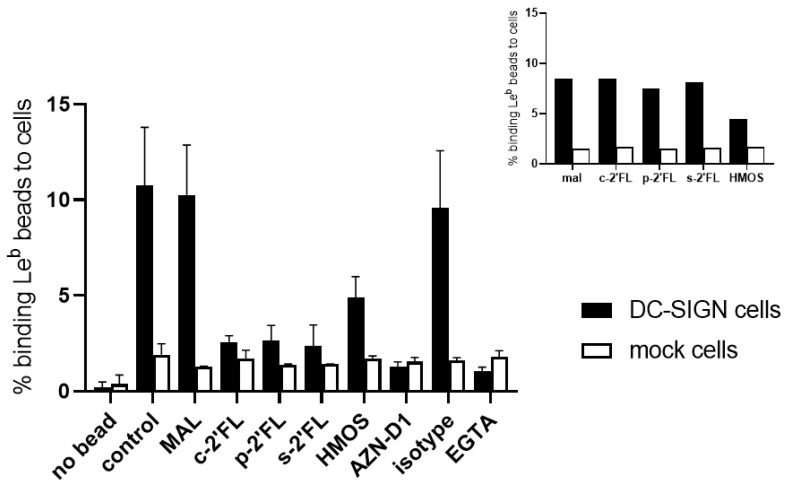
Cell-ligand competition assay with the DC-SIGN expressing cells and mock cells and c2′-FL, p2′-FL, s2′-FL, HMOS, maltotriose (MAL, negative control of the 2′-FLs and HMOS), isotype antibody (negative control), AZN-D1 (DC-SIGN binding antibody, positive control), and EGTA (as the inhibition control). At a 5 mg/mL concentration of c2′-FL, p2′-FL, and s2′-FL, the HMOS inhibits the binding of the Le^b^ coated fluorescent beads to the DC-SIGN expressing cells. The insert shows the inhibition effect in the 1 mg/mL concentration of c2′-FL, p2′-FL, s2′-FL, HMOS. Results are presented as the mean % of the cells binding the Le^b^- coated fluorescent beads +/- SD, the data from two separate experiments were performed in duplicate (n = 4 technical replicates). For the insert, n = 2 technical replicate. c-2′-FL = biotechnologically produced commercially available 2′-FL, p2′-FL = purified c-2′-FL, and s2′-FL = chemically synthesized 2′-FL.

A cell-ligand competition assay was performed, using the DC-SIGN expressing OUW cells, using the Le^b^-coated fluorescent beads as a multivalent bona fide ligand. The anti-DC-SIGN mAbs AZN-D1 was used as a positive control to block the cellular interactions between DC-SIGN and the Le^b^-coated fluorescent beads. An isotype antibody was used as a negative control to differentiate the non-specific background signals. The DC-SIGN specific blocking antibody, AZN-D1, as well as EGTA, inhibited the binding of the Le^b^-coated fluorescent beads to the DC-SIGN expressing cells. These results indicate a specific binding of Le^b^ to DC-SIGN on the DC-SIGN-expressing cells. The DC-SIGN expressing cells binding to Le^b^ were inhibited by c2′-FL, p2′-FL, s2′-FL, and HMOS at a concentration of 5 mg/mL (Figure 3). Here, we observe again that 2′-FL can mimic the inhibitory effect of the HMOs, which is a mixture of various complex oligosaccharides. However, this effect was not observed at the 1 mg/mL concentration of all 2′-FLs (Figure 3 insert). All 2′-FLs lost the efficiency of inhibition of the binding of Le^b^ to the DC-SIGN expressing cells, but the HMOS (1 mg/mL) retained some of the inhibition at this concentration.

### 2.3. Molecular Dynamic Simulation Suggested That 2′-FL Exists in Bioactive Conformation to Bind DC-SIGN but Not Langerin

**Figure 4:**
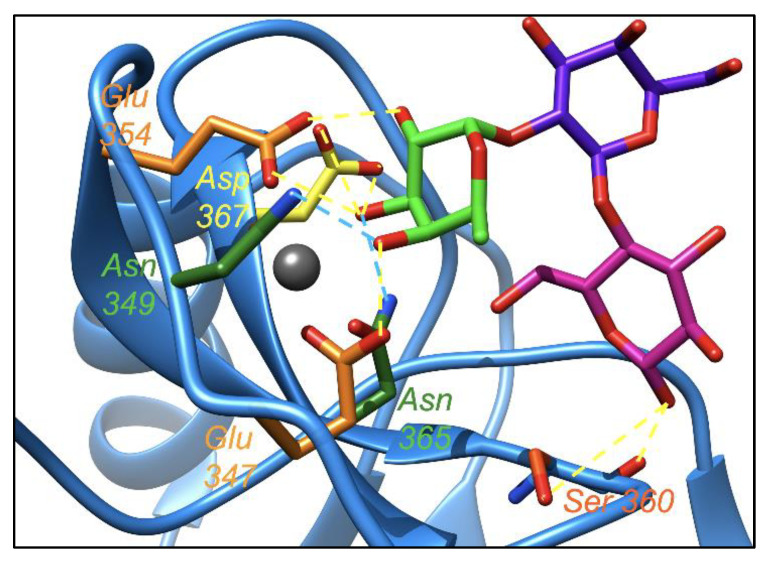
The hydrogen-bonding (dotted lines) network of 2′-FL and DC-SIGN. DC-SIGN interacting domain is represented as a blue ribbon. Fucose is coloured in green; galactose is coloured in dark blue; glucose is coloured in magenta; Ca^+2^ is coloured as a grey sphere. All of the amino acids’ sidechain colours are matched with their respective label colours. The yellow dotted line represents interactions of 2′-FL as a hydrogen bond donor. The blue dotted line represents the interactions of 2′-FL as a hydrogen bond acceptor.

For the first time, an MD simulation study was performed to obtain a greater insight into the binding mode of DC-SIGN with 2′-FL. The simulation showed that the terminal fucose in 2′-FL acts both as a hydrogen bond donor and acceptor (Figure 4). It remains hydrogen-bonded with DC-SIGN most of the time throughout the duration of the simulation (400 ns) (see Table S1 for detailed interactions). The terminal fucose in 2′-FL remains hydrogen-bonded with DC-SIGN most of the time with the glutamic acid (354 and 347) and aspartic acid (367) of DC-SIGN, via the hydrogen of -OH of fucose at 2, 4, and 3 positions, respectively (as a hydrogen bond donor). The glucose anomeric -OH (as a hydrogen bond donor) showed a connection to the backbone oxygen and sidechain oxygen of the serine (360) of DC-SIGN. The asparagine (365 and 349) of DC-SIGN was hydrogen-bonded via the oxygen of -OH of fucose at 3 and 4 positions, respectively (as hydrogen bond acceptor). All of the hydrogen bonding interactions are shown in Figure 4 and Table S1.

The analysis of the binding mode and the dihedral angle of 2′-FL were performed to gain insight into its flexibility. 2′-FL remained quite rigid when it was bonded and non-bonded to DC-SIGN, suggesting that 2′-FL preferentially displayed the bioactive conformation (pre-organized) before binding (Figure S2) to DC-SIGN.

The binding affinity or stability of a complex depends on many factors. Non-covalent interactions, such as hydrogen bonds, contribute to stabilizing the complex of the protein and its ligand. Preorganized ligands also form the complex with its receptors with a better affinity or stability, due to its less entropic penalty (please see the section entitled “Explanation of the Possible Better Binding of 2′-FL to DC-SIGN” in SI for the detailed explanation). As 2′-FL is preorganized and forms a hydrogen-bonding network with DC-SIGN, the affinity or stability of the complex of DC-SIGN and 2′-FL will be better than other flexible ligands. Using, MMPBSA we calculated the enthalpy of the interaction of 2′-FL and DC-SIGN, and that is 0.6 ± 0.4 Kcal/mol.

The MD simulation with 2′-FL and langerin revealed that there was no binding between them. 2′-FL left the binding site of langerin after 200 ns. When a ligand leaves the binding pocket, it means the molecule has less energy (Gibbs) in the free state than in the bound state. Thus, the binding between the two entities may be very weak or none.

**Figure 5:**
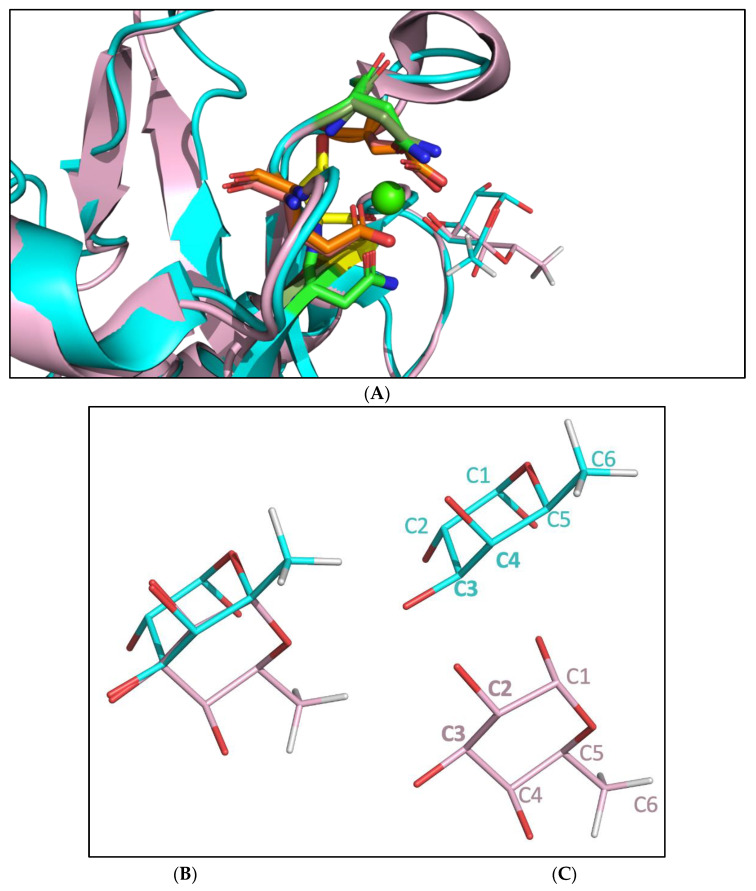
(**A**): Both DC-SIGN (cyan) and langerin (light pink) have been aligned. The residues interacting through the calcium ion (green ball) are displayed in sticks and fucose residues in the wire representation. Aspartic acid is shown in yellow, asparagine is shown in green, and glutamic acid is shown in orange. Both proteins show the same residues and the same geometry on the binding site as can be observed in the superimposed image. (**B**): Superimposition of the two fucose poses, is shown to depict the significant orientation difference between the two fucoses. The hydrogens have been hidden in the figure, for clarity purposes, only the methyl group hydrogens are shown for a clear depiction of the orientation differences between the two fucoses. Only the fucose part of the whole ligands (LNFP III for DC-SIGN and blood group B trisaccharide for langerin) is shown here. (**C**) The two fucose poses are shown separately with the atom numbers, for clarity.

We compared the binding pockets of DC-SIGN and langerin to understand the difference in the binding by 2′-FL (Figure 5). Both proteins show a comparable CRD, and in the superimposition, it can be observed that both structures are very similar, containing the same residues: two glutamic acids, two asparagines, and one aspartic acid (Figure 5A). However, fucose utilizes hydroxyl groups hanging from C3 and C4 to interact with the calcium ion in the recognition of the DC-SIGN (cyan), while the hydroxyl groups linked to the C2 and C3 are employed by the fucose in the interaction with the langerin (light pink) through the calcium ion (Figure 5B,C). This binding mode difference of the fucose forces the ligand to orientate the rest of the ligand in a different way.

This difference in the orientation of fucoses results in differences in some of the key hydrogen bonding interactions. In the case of DC-SIGN and 2′-FL interactions, the hydrogen bonding interactions of Glu347 and Glu 354 (as a hydrogen bond acceptor) with fucose O4 and fucose O2 (as a hydrogen bond donor), respectively appear throughout the whole trajectory (98% and 99 % of the trajectory time, respectively) of the MD simulation. Another hydrogen bonding interaction between Asp 367 (as a hydrogen bond acceptor) with fucose O3 (as a hydrogen bond donor) is present for 40% of the MD simulation trajectory of the DC-SIGN and 2′-FL interaction. In the case of the langerin and 2′-FL interaction, due to the different orientations of fucose, the hydrogen bonding interactions in the binding pocket of langerin occurred for a shorter time in the MD simulation trajectory (see Table S2. The interaction between Glu 293 (as a hydrogen bond acceptor) and fucose O3 (as a hydrogen bond donor) is present for 50% of the simulation trajectory. Another interaction between Glu 285 (as a hydrogen bond acceptor) and fucose O2 (as a hydrogen bond donor) is present for 42% of the simulation trajectory. The interaction between fucose O2 (as a hydrogen bond acceptor) and Asn 287 (as a hydrogen bond donor), is only present for 11% of the simulation trajectory.

### 2.4. Le^b^ Interacts with DC-SIGN with Two Fucoses Operating One at a Time: Consequences for the Displacement by 2′-FL

**Figure 6:**
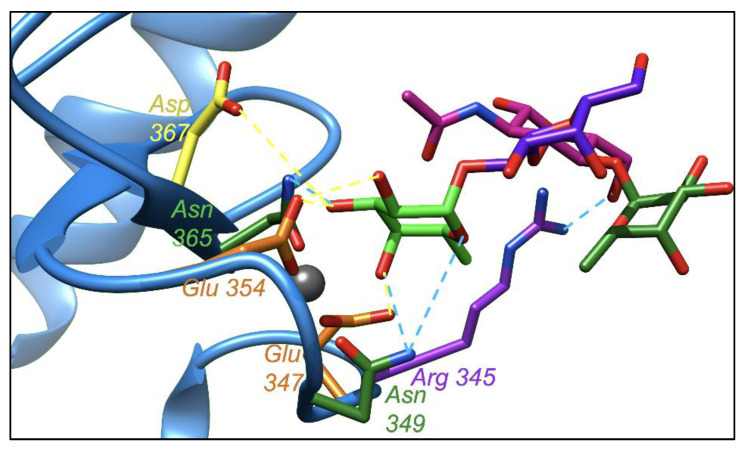
The hydrogen bonding (dotted lines) network of Le^b^ and DC-SIGN (the bonded fucose is linked to the galactose). DC-SIGN interacting domain is represented as a blue ribbon. Fucose is coloured in green; galactose is coloured in dark blue; N-Acetylglucosamine is coloured in magenta; Ca^+2^ is coloured as a grey sphere. All of the amino acid sidechain colours are matched with their respective label colours. The yellow dotted line represents the interactions of Le^b^ as a hydrogen bond donor. The blue dotted line represents the interactions of Le^b^ as a hydrogen bond acceptor.

In Le^b^, one fucose is connected with galactose while the other fucose is connected with N-acetylglucosamine. The MD simulation showed that Le^b^ binds DC-SIGN with its two fucoses in two different ways. These two different binding modes appeared one at a time. When Le^b^ binds to DC-SIGN via its fucose connected to galactose, the following hydrogen-bonding networks were observed in the MD simulation (see Table S3 for detail). Most of the time, the glutamic acids (347 and 354) and aspartic acid (367) of DC-SIGN were connected to fucose (which is connected to galactose) via the hydrogen of -OH of fucose at 4, 2, and 3 positions, respectively (as a hydrogen bond donor). The asparagine residues (349 and 365) of DC-SIGN were connected via the oxygen of -OH of fucose at 4 and 3 positions, respectively (as a hydrogen bond acceptor). The hydrogen bonding connection between N-acetylglucosamine via O6 and arginine (345) of DC-SIGN was also observed. The fucose ring oxygen was also connected with asparagine (349). The interactions are shown in Figure 6.

The other binding mode of Le^b^ with fucose which is connected to N-acetylglucosamine, is described in Figure S3.

The MD trajectory analysis showed an important hydrogen-bond network established between Le^b^ and DC-SIGN. These interactions were comparable with the interaction network found for 2′-FL. However, Le^b^ displayed a higher flexibility in water than 2′-FL in the free state and more than one conformation co-exists in the solution (Figure S2). The RMSD calculated over the heavy atoms along the 1-microsecond trajectory, showed the coexistence of more than one conformation in the case of Le^b^ and just one conformation for 2′-FL (Figure S4). This increased freedom of the dihedral angles contributes to augmenting the entropy penalty, which may diminish the binding affinity of this ligand to DC-SIGN. Using MMPBSA, we calculated the enthalpy of the interaction of Le^b^ and DC-SIGN. The enthalpy is 0.5 ± 0.4 Kcal/mol when Le^b^ is interacting with fucose connected to galactose. The enthalpy is 7.8 ± 0.7 Kcal/mol when Le^b^ is interacting with fucose connected to GlcNAc.

The Ramachandran-like plot (Figure S5) showed that 2′-FL adopted predominantly the bioactive conformation. However, Le^b^ showed mostly a restricted bioactive conformation in the bound state, although with a higher degree of flexibility than 2′-FL in the free state. The highly restricted conformation in the bound state for Le^b^ and its larger degree of flexibility in the free state cause a higher entropic penalty for binding to DC-SIGN than that of 2′-FL. Thus, Le^b^ may bind to DC-SIGN with a lower affinity than 2′-FL.

2′-FL shows a similar binding enthalpy to Le^b^, when Le^b^ is interacting with fucose connected to galactose. There is a significant difference, however, in the enthalpy of the interaction when Le^b^ is interacting with fucose connected to N-Acetylglucosamine. The lower binding enthalpy of 2′-FL suggests that it may bind to DC-SIGN with a higher affinity than that of Le^b^. As the two binding modes of Le^b^ operate one at a time, while switching the binding mode, there may be an opportunity for 2′-FL to enter into the binding pocket of DC-SIGN.

## 3. Discussion

In line with previous findings, we have shown that 2′-FL binds to DC-SIGN but not to langerin in the tested experimental conditions. In addition, our data show that in both the ligand-receptor competition assays and the cell-ligand competition assays, 2′-FL competes with the DC-SIGN ligands fucose monosaccharide and Le^b^, for binding to DC-SIGN in a dose-dependent way. It is worth mentioning that 2′-FL can compete with the DC-SIGN ligands fucose monosaccharide and Le^b^, even though these ligands were represented on the PAA bead as multivalent ligands. Moreover, the observed inhibitory effect of 2′-FL on the DC-SIGN ligand binding is comparable to that of a total HMO mixture.

Recently, it has become possible to produce 2′-FL, biotechnologically on a large scale [24]. It is unclear whether the 2′-FL immunomodulatory effects are due to the HMO itself or rather due to the presence of side products [27]. Earlier work indicated that the bacterial production of bioactive proteins, such as heat shock proteins or other endogenous proteins, might have immunomodulating effects linked to the microbial contaminants, rather than the actual protein [28]. Similar effects are also published on the immune effects of HMOs, whereupon with the rigorous purification of the HMOs, the immune effects were significantly reduced [29]. However, recent work by Sodhi et al. [17], using experimental and modelling techniques, showed that 2′-FL can bind to TLR4 and inhibits its activation by its ligand lipopolysaccharide (LPS), suggesting that 2′-FL might indeed directly bind to the host receptors. Here we show that all forms of 2′-FL, either synthetically or biotechnologically produced, as well as purified, can compete with fucose or Le^b^ for binding to DC-SIGN. Our results show that the possible side products in the biotechnologically produced 2′-FL are not playing a role in the 2′-FL-induced inhibition of the binding of the prototypical ligands to DC-SIGN.

There is a concentration difference in the effects of 2′-FL on the DC-SIGN-ligand interactions assessed by the ligand-receptor and cell-ligand competition assays. In the ligand-receptor competition assay, the effective concentration of all forms of 2′-FLs is 1 mg/mL (2 mM). This concentration is similar to the previously reported, where it was also shown that IC_50_ of 2′-FL towards the DC-SIGN binding is in the physiological concentration (1 mM) [25]. In addition, in human milk, the typical physiological concentration of 2′-FL is 0.5–2.5 g/L (1–5 mM) [25]. In the cell-ligand competition assays, however, the concentration of 2′-FL needed to inhibit the DC-SIGN-Le^b^ interaction is higher (10 mM).

Our MD simulation data supports the insight that 2′-FL directly binds to the CRD of DC-SIGN. 2′-FL remains mostly hydrogen-bonded with DC-SIGN during the entire simulation time (400 ns). 2′-FL exists in a bioactive conformation before binding to DC-SIGN, whereas Le^b^ has a flexible structure before binding to DC-SIGN. Moreover, 2′-FL and Le^b^ have a comparable binding enthalpy when Le^b^ is interacting with fucose connected to galactose. However, Le^b^ has a significantly higher binding enthalpy when Le^b^ is interacting with fucose connected to N-acetylglucosamine. Due to the pre-existence of the bioactive conformation, 2′-FL binds to DC-SIGN with a less entropic penalty and its lower binding enthalpy suggests that the binding affinity of 2′-FL to DC-SIGN may be stronger than that of Le^b^. Therefore, 2′-FL can inhibit the binding of Le^b^ to DC-SIGN. Furthermore, Le^b^ has two binding modes, one via fucose connected to galactose and another via fucose connected to N-acetylglucosamine. These binding modes are in action one at a time. This suggests that Le^b^ may leave the binding pocket of DC-SIGN while changing its binding mode and making room for other ligands, such as 2′-FL, to bind. The presence of two fucoses does not add advantages to Le^b^ over 2′-FL for binding more strongly to DC-SIGN. Le^b^ is not large enough to interact with the binding pockets of two DC-SIGN molecules at the same time. Hence, strengthening the binding capacity of Le^b^ through multivalency is not possible.

The MD simulation data regarding the interaction between langerin and 2′-FL demonstrated that 2′-FL leaves the binding pocket of langerin after 200 ns, suggesting that 2′-FL does not bind to langerin. The comparison of the binding pockets of DC-SIGN and langerin while interacting with their fucosylated ligands (LNFP III for DC-SIGN and blood group B trisaccharide for langerin) shows that the interacting fucose moiety of the ligands utilizes different atoms to interact with the calcium ion. This alteration in the protein-ligand communication forces the ligands to adopt distinct orientations. Thus the presence of the hydrogen bonding interactions in the MD simulation trajectory differed. Langerin showed much weaker hydrogen bonding interactions with 2′-FL in the MD simulation trajectory.

In general, the relative binding affinity of the Lewis antigens to DC-SIGN-Fc is of the following orders: Le^b^ (47%) > Le^y^ (45%) > Le^a^ (34%) > Le^x^ (22%) [30]. The K_d_ value of Le^x^ (presented on the multivalent PAA constructs) is 1.74 µM [30]. In our study, we demonstrated that 2′-FL replaces Le^b^ (presented on the multivalent PAA constructs), which has the strongest affinity to DC-SIGN amongst the Lewis antigens [30]. Therefore, 2′-FL can replace other Lewis antigens or fucosylated ligands, which are more flexible than 2′-FL and have a similar or less binding affinity, from the CRD of DC-SIGN.

This study implicates that 2′-FL binds to DC-SIGN but not to langerin and 2′-FL is able to outcompete the Lewis antigens for binding to the CRD of DC-SIGN. Based on the importance of the Lewis antigens in the host-microbe interactions [31] at the mucosal surface, as well as the anatomical localization and expression of DC-SIGN on the myeloid subsets of the immune cells, these interactions might be of importance in shaping the microbiota and immune balance in health and disease [32].

## 4. Materials and Methods

### 4.1. Antibodies and Beads

TSA buffer: 5 mL 10× TSM + 0.5 gr BSA (Millipore, 126575, Lot 3134664) + 45 mL Milli-Q (ppb < 2) BSA: (Sigma-Aldrich, lyophilized powder, ≥96%, agarose gel electrophoresis), HBSS: Gibco; EGTA, Sigma-Aldrich, E3889; AZN-D1: isolated, as described in ref [33]: anti-human horseradish peroxidase: Jackson; mouse IgG1 (isotype control): Invitrogen Lot 4347632, clone P3.6.2.8.1 cat 16-4714-82; 3,3′,5,5′-Tetramethylbenzidine, TMB, Sigma-Aldrich 860336; Streptavidin: Sigma S4762, Lot #098M4008V; Tween 20, Sigma-Aldrich, P1379; α-L-fucose-PAA beads and Le^b^-PAA beads: Lectinity, heck; TransFluoSpheres carboxylate-modified microspheres: 488/645 nm, 1.0 μm; Molecular Probes, Eugene, OR.

### 4.2. Cells

The DC-SIGN expressing cells (OUW-SIGN cells) are generated by the transduction of EBV-BLCL with DC-SIGN, as previously described [34,35].

### 4.3. HMOs

c2′-FL and whole HMOS were generously donated by Danone Nutricia, The Netherlands. p2′-FL was made by purifying c2′-FL. The synthetic 2′-FL (s2′-FL) was purchased from Carbosynth (www.carbosynth.com). The c2′-FL contains 94.1 % 2′-FL, 1.4% 3-FL, 1.5% DFL, 2.6% fucosyl-galactose, 0.1% fucose (measured by HAPEC-PAD). The s2′-FL contains 95% 2′-FL (purity min 95% by ^1^H NMR as mentioned by Carbosynth). The component analysis of HMOS was not carried out.

### 4.4. Purification of c2′-FL to Obtain p2′-FL

One gram of c2’-FL was dissolved in 10 mL of MilliQ water. The solution was poured into a separatory funnel. The solution was extracted with chloroform (3 × 5 mL). Then 300 mg of activated charcoal (pre-washed with MilliQ water) was added to the solution and left for 1 hr with occasional stirring. The charcoal was filtered with Whatman filter paper and finally with 2 µM pore-sized filter paper. Then the water layer was lyophilized to collect 800 mg of p2′-FL (which contains mostly 2′-FL with a trace of 3-FL and DFL, as observed in MALDI-MS).

### 4.5. Ligand-Receptor Competition Assay

The Polyacrylamide polymers (PAAs) functionalized with different glycans were purchased from Lectinity, heck MW approximately 20 KDa, carbohydrate content, around 20% mol. Fifty µL of Le^b^-PAA or fucose-PAA at 1.0 µg/mL in PBS (10 mM, pH = 7.4) were used to coat the Nunc MaxiSorp plates overnight at room temperature. Then, after discarding and washing with PBS (2 × 200 µL), the wells were blocked with 200 µL of 1% BSA (Sigma-Aldrich, lyophilized powder, ≥96%, agarose gel electrophoresis) in HBSS containing CaCl_2_/MgCl_2_ (Gibco) at 37 degrees for 30 min. In parallel, DC-SIGN-Fc (5 µg/mL or 0.5 µg/mL or 0.1 µg/mL) or langerin-Fc (5 µg/mL) was pre-incubated (competition) with serial dilutions of each c2′-FL p2′-FL, s2′-FL, and HMOS in an assay buffer (1% BSA in HBSS), for 30 min at 37 degrees. Both chimeric constructs, DC-SIGN-Fc and langerin-Fc, consist of their extracellular domains fused to the Fc portion of human IgG1. They were produced from established transfectants as described previously [36,37,38].

The blocking solution was discarded from the plate wells, and the mixture of DC-SIGN-Fc (at concentrations of 5.0, 0.5, or 0.1 µg/mL) and each of c2′-FL, p2′-FL, s2′-FL, or HMOS (at concentrations of 1.0, 10, 100, and 1000 µg/mL) in an assay buffer (1% BSA in HBSS) were added to the plate coated with Le^b^-PAA or fucose-PAA.

Then, after 2 h at room temperature, the wells were washed with 0.05% Tween-20 in HBSS (3 × 200 µL) and then 50 µL of the anti-human horseradish peroxidase (0.5 µg/mL, Jackson, Goat anti-Human IgG-HRP) was added. Then, after 30 min at room temperature, the wells were washed with 0.05% Tween-20 in HBSS (4 × 200 µL).

Finally, 100 µL of the substrate solution (3,3′,5,5′-Tetramethylbenzidine, TMB, in citric/acetate buffer, pH = 4, and H_2_O_2_) was added and after 5 min of incubation at room temperature, the reaction was stopped with 50 µL of H_2_SO_4_ (0.8 M) and the optical density was measured at 450 nm in an ELISA reader. The experiment was performed in duplicate, and the data were normalized over the signal at 450 nm from the non-coating wells. Data were expressed as the % of the binding and they were normalized over the OD signal from the wells without any competition (i.e., the binding between the receptors and the positive control PAA-Fuc without competitors and without prior incubation with the studied molecules). This OD value was set as 100% of the interaction.

### 4.6. Fluorescent Bead Adhesion Cell-Ligand Competition Assay

To demonstrate the effect of 2′-FLs and HMOS on the binding of Le^b^ to the whole cells, a fluorescent bead adhesion assay was used, as previously described [39]. TransFluorSpheres were covalently coupled to streptavidin. Then the biotinylated PAA-Le^b^ beads were coupled to the streptavidin beads. Briefly, 7.5 μL of the streptavidin beads (+/− 8.25 × 10^7^ beads) and 10 μL solution of the biotinylated PAA-Le^b^ (of 1 mg/mL concentration) were taken in an Eppendorf and 300 μL of PBA was added into that. The mixture was incubated at 37 degrees for 2 h, rotating at 550 rpm. Following the incubation, the mixture was centrifuged at 13,000 rpm using an Eppendorf centrifuge. The beads were washed 2× using 500 μL PBA. The beads were suspended in 50 μL PBA (+/− 8.25 × 107 beads/50 μL).

The DC-SIGN expressing cells (OUW-SIGN) and the mock cells were made at a cell concentration of 10^6^ cells/mL in TSA. Then, we put 50 μL/well of the cells into a 96-well V-bottom plate (50.000 cells/well). Then, each of c2′-FL, p2′-FL, s2′-FL, and HMOs were added, either in 5 mg/mL or 1 mg/mL, as the final concentration. EGTA (final concentration 10 mM), the isotype antibody (final concentration 25 µg/mL), and AZN-D1 (final concentration 25 µg/mL) were added into the well. Following the addition of all of the components, the plate was incubated for 15 min at 37 °C. Then, 10 μL/well Le^b^-PAA-beads (final 1 μL beads/well) were added. Then, the plate was again incubated for 30 min at 37 °C. The FACS acquisition on Fortessa was performed immediately and the cells were put on ice. The beads were measured in channel blue A-A at a voltage of 400. In total 30,000 events were recorded.

### 4.7. Molecular Dynamics Simulation

The interactions between DC-SIGN and the different ligands, 2′-FL, Le^b^ were further evaluated in molecular dynamic simulations. In the case of Le^b^, two trajectories were run to analyze the recognition process, one trajectory for each of the two fucoses that this compound possesses. Oligosaccharide structures, both 2′-FL and, Le^b^, were obtained by employing the Carbohydrate Builder tool on the website Glycam.org [40]. The DC-SIGN and ligand complexes were built using the crystal structure (PDB id: 1sl5) where DC-SIGN is complexed with the ligand LNFP III (fucosylated ligand). The fucose in the ligand serves as a model for the orientation of the different ligands. The cycle-forming atoms, together with the hydroxyl linked to the anomeric carbon, were selected to align the ligands (2′-FL and Le^b^). Following the superimposition of the ligands, the original ligand (LNFP III) was removed to obtain the protein-ligand complex coordinates. The superimposition of the starting structures is shown in Figure S6. The complex was solvated with the water model TIP3P [41] in a 10 Å cubic box. The simulations were carried out using the AMBER 18 suite [42], the protein parameters were taken from the ff14SB [43] force field and the glycan moiety from the GLYCAM_06j-1 force field [44]. In the case of langerin, the complex was built using the crystal structure of the langerin RBD, in a complex with the blood group B trisaccharide (PDB id: 3P5G). The blood group B trisaccharide was replaced by 2′-FL prior to running the simulation. The protocol for the MD simulations applied to the molecules in the bound state, starts with a two-step minimization process: first, the solute was fixed, and only the solvent molecules were allowed to move; and during the second step, all of the atoms were allowed to be minimized in the simulation cell. Then, a three-step molecular dynamic simulation was performed, and the temperature was raised from 0 to 300 K, under the constant pressure of 1 atm and the periodic boundary conditions. Harmonic restraints of 10 kcal·mol^−1^ were applied to the solute, and the Berendsen temperature coupling scheme [45] was used to control and equalize the temperature. The time-step was kept at 1 fs during the heating stage. The water molecules were treated with the SHAKE algorithm, such that the angle between the hydrogen atoms remained fixed. The particle-mesh-Ewald (PME) method was employed to model the long-range electrostatic interactions [46]. An 8 Åcut-off was applied to the Lennard–Jones and electrostatic interactions. Each system was equilibrated for 100 ps with a 2 fs timestep at a constant volume and temperature of 300 K. The production trajectories were then run for an additional 400 ns under the same simulation conditions. The protocol followed the performance of the MD simulation on the glycans in the free state, which is similar to the former one (bound state), but the production trajectory was extended up to 1 microsecond. Cpptraj module of AMBER 18 was employed to process the trajectory. MMPBSA was employed to calculate the enthalpy values. Fifty frames evenly separated among the last 5000 steps of the trajectory were considered for the enthalpy calculation.

## Data Availability

The data underlying this article are available in the article and in the online Supplementary Materials.

## Supplementary Materials

The following supporting information can be downloaded at: https://www.mdpi.com/article/10.3390/ijms232314745/s1.

## Abbreviations

CRD: Carbohydrate recognition domain; CLR: C-type lectin receptor; DC-SIGN: Dendritic Cell-Specific Intercellular Adhesion Molecule-3- Grabbing Non-integrin; EBV-BLCL: Epstein–Barr virus-transformed B-lymphoblastoid cell line; ELISA: Enzyme-Linked Immunoassay; FACS: Fluorescence-activated cell sorting; Le^b^: Lewis B; HBSS: Hank’s balanced salt solution; HMOs: Human milk oligosaccharides; 2′-FL: 2′-Fucosyllactose; EGTA: Egtazic acid; MD: Molecular Dynamics; PME: Particle-mesh-Ewald; mAbs: monoclonal antibodies; TMB: 3,3′,5,5′-Tetramethylbenzidine; TSM: Tris-Saline with Magnesium
